# Complete Genome Sequence of a *Gamaleyavirus* Phage, Lytic against Avian Pathogenic Escherichia coli

**DOI:** 10.1128/mra.00896-22

**Published:** 2022-10-26

**Authors:** Madina S. Alexyuk, Andrey P. Bogoyavlenskiy, Pavel G. Alexyuk, Kuralay S. Akanova, Yergali S. Moldakhanov, Adolat Manakbayeva, Vladimir E. Berezin

**Affiliations:** a Research and Production Center for Microbiology and Virology, Laboratory of antiviral protection, Almaty, Kazakhstan; Portland State University

## Abstract

We report the complete genome sequence of the lytic bacteriophage vB_EcoS_Uz1, which was isolated from wastewater near Almaty, Kazakhstan using the avian pathogen Escherichia coli host. Its complete genome is 72,583 bp in length, with a GC content of 43%. vB_EcoS_Uz1 belongs to the Gamaleyavirus genus of the *Caudoviricetes* class.

## ANNOUNCEMENT

Amid the high incidence of antibiotic-resistant E. coli strains in the poultry industry (1), it is necessary to develop alternative antibacterial preparations that have a minimal effect on the host organism. One such possibility is the use of bacteriophages to control colibacillosis. Several studies have shown that bacteriophages can be used to prevent and treat colibacillosis in poultry (2–4).

vB_EcoS_Uz1 was isolated by enriching 10 mL filtered municipal wastewater collected near Almaty in 2021 with 1 mL (10^8^ cells/mL) avian pathogenic E. coli isolate (F.H.pr. Uz), adding 1 mL 10x Nutrient broth #2 (NB) and incubating the sample for 18 h at 37°C. After incubation, the enriched sample was centrifuged at 6000 rpm and filtered through 0.45-μm PES syringe filters. The filtrate was tested for phage activity using double agar overlay method. The phage was purified by 3 cycles of isolation of single plaques propagated on the strain F.H.pr.Uz in a NB #2 at 37°C. Phage lysate was generated at a multiplicity of infection (MOI) ratio of 1, followed by filtration and sedimentation of phages by centrifugation at 100,000 × *g* for 90 min (5). The obtained pellet was dissolved in a minimum volume of PBS and treated with RNase A and DNase to remove bacterial DNA and RNA. Phage genomic DNA was isolated using a PureLink viral DNA/RNA minikit (Thermo Fisher Scientific) and sequenced using a Nextera XT DNA library preparation kit (Illumina). Libraries were sequenced on an Illumina MiSeq instrument (2 × 300-bp paired-end reads) with an average coverage depth of 120-fold. FastQC v0.11.5 (https://www.bioinformatics.babraham.ac.uk/projects/fastqc) was used for read quality control. Low-quality reads were filtered out and adapters trimmed with Trimmomatic (6) from the Genome Detective tool (7). The average read length after trimming was 286 bp. As a result, 1,035,661 reads were assembled using SPAdes 3.12.0 (8). A contig of 72,583 bp was obtained. The virus genome was reassembled *de novo* using raw reads and the contig. Afterwards, the raw sequences were mapped to the assembled genome. The presence of sequences with a higher level of coverage at the ends and identical fragments of 469 bp on the left and right sides of the genome confirmed its physical ends. ORFs were predicted using the ORF finder tool (https://www.ncbi.nlm.nih.gov/orffinder/) and Geneious Prime. tRNA coding regions were identified with tRNAscan-SE (9). All tools were run with default parameters.

**FIG 1:**
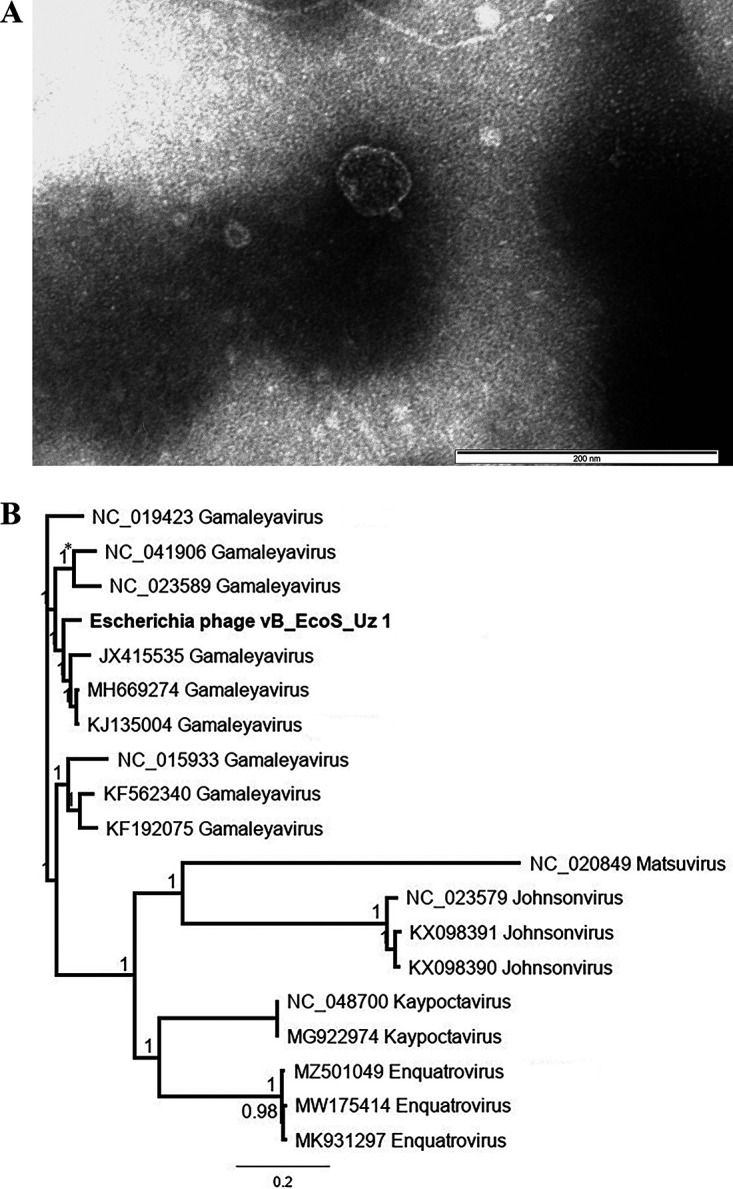
(A) Transmission electron micrograph of vB_EcoS_Uz1 (phage sample was prepared for imaging as in reference [10]. In brief: 3% phosphotungstic acid [pH 6,8] was used to stain the sample, the grids were observed with a JEM-2100 “JEOL” [Japan]). (B) Phylogenetic tree generated using FastTree Plugin default settings in Geneious Prime based on the whole-genome sequences. * **-** indicates the converse of the topological (‘‘Robinson-Foulds’’) distance. All selected phages were obtained from ICTV 2021 Master Species List (MSL37) (https://ictv.global/msl).

vB_EcoS_Uz1 has an icosahedral capsid (Fig. 1A), possesses a circular, double-stranded DNA genome of 72,583 bp in length, with a GC content of 43%. Genome annotation predicts 97 coding features, of which 37 have assigned functions, including 2 tRNAs (Xle, Cys). The phylogenetic tree constructed using FastTree Plugin 2.1.11 based on the whole-genome sequences indicates that its closest relatives are Enterobacteria phage Bp4 (KJ135004), Escherichia phage PD38 (MH669274) and Escherichia phage ECBP1 (JX415535) (Fig. 1B), and establishes its belonging to the Gamaleyavirus genus.

### Data availability.

The vB_EcoS_Uz1 genome is available in GenBank with accession number OP312987. The raw sequence reads are available at NCBI SRA database with accession number SRR21162442.
